# Predictors of health literacy among Deaf American Sign Language users

**DOI:** 10.1016/j.pec.2025.109348

**Published:** 2025-09-16

**Authors:** Michael M. McKee, Melissa Plegue, Sara Champlin, Joseph Hill, Tiffany Panko, Lorraine R. Buis, Ananda Sen, Michael K. Paasche-Orlow, Peter C. Hauser

**Affiliations:** a Department of Family Medicine, University of Michigan Medical School, Ann Arbor, MI 48104, USA; b School of Journalism, University of North Texas, Denton, TX 76201, USA; c Center for Black Deaf Studies, Gallaudet University, Washington, DC 20002, USA; d National Technical Institute for the Deaf, Rochester Institute of Technology, Rochester, NY 14623, USA; e Division of General Internal Medicine, Department of Medicine, Tufts University School of Medicine and Tufts Medical Center, Boston, MA 02116, USA; f Department of Biostatistics, University of Michigan School of Public Health, Ann Arbor, MI 48109, USA

**Keywords:** Deaf, Sign Language, ASL, Health Literacy, Race, People of Color

## Abstract

**Objective:**

Health literacy is an important predictor of individuals’ health, medical adherence, and health-related decision making. This study investigated the predictors of health literacy among Deaf American Sign Language (ASL) users.

**Methods:**

408 Deaf ASL users and 445 Hearing English speakers were administered the Newest Vital Sign, a measure of health literacy available in both English and ASL, along with assessments of language proficiency and reading skills.

**Results:**

Deaf participants had 3.7 times greater odds of inadequate health literacy (95 % CI: 2.7, 4.9) compared to their hearing counterparts. Binary logistic regression revealed that Deaf participants’ ASL proficiency and English reading grade equivalent explained 34.2 % (Nagelkerke *R^2^*) of the variance in health literacy, χ^2^(2) = 101.520, *p* < .001. Among hearing participants, by contrast, English proficiency and English reading grade equivalent explained 47.8 % of the variance in health literacy, χ^2^(2) = 171.071, *p* < .001.

**Conclusions:**

Deaf people are at risk for having greater difficulty to find, understand, and use information and services to inform health-related decisions and actions for themselves and others.

**Practice implications:**

Health professionals and health systems should allocate resources to mitigate health literacy barriers among Deaf people and make health information more available in ASL.

## 1. Background

Health literacy is the degree to which individuals have the capacity to obtain, process, and understand basic health information and services needed to make appropriate health decisions [1,2]. Health literacy is a better predictor of some health outcomes, such as health status, adverse medical outcomes, ineffective management of chronic disease, than race, ethnicity, education, and socioeconomic status [3–6]. Inadequate health literacy is a common barrier in patient education, and those with inadequate health literacy often have less disease and treatment knowledge, greater difficulty adhering to medical recommendations, more challenges navigating health care systems, worse health status, and higher rates of mortality [7–12].

Deaf American Sign Language (ASL) users (thenceforth, *Deaf signers*) represent approximately 500,000 individuals in the USA who do not have access to a spoken language [13,14]. This cultural and linguistic group faces widespread social, language, and information marginalization, impacting their health care access and quality of care [15–21]. Deaf signers frequently encounter inaccessible health care, often through failure to provide necessary accommodations (e.g., sign language interpreters) and ongoing stigma [16,17,20,21]. They rarely receive language concordant health care services, placing them at high risk for miscommunication with their health providers [19,22–24]. McKee and his colleagues (2015) adapted, translated and validated an ASL version of the *Newest Vital Sign* [5] measure of health literacy (*ASL-NVS*). Deaf adults were found to be 6.9 times more likely than their hearing peers to have inadequate health literacy. The study did have inherent limitations, including generalizability concerns due to recruitment from a single metropolitan area and the lack of data regarding audiometry, language proficiency, and reading skills.

Past studies have also demonstrated that People of Color were at a greater risk of having inadequate health literacy [3,25,26] than White respondents, and that health literacy was found to be a better predictor of health status than their race or educational attainment [3,26]. It is unknown if intersectional factors compound the risk of inadequate health literacy for Deaf signers.

The present study is a follow-up investigation of Deaf signers’ health literacy from a broader sample with confirmed severe or profound hearing loss who use ASL or both ASL and English to communicate. The objective was to investigate the predictors of health literacy among Deaf signers to demonstrate the capacities they bring to their medical appointments and self-care and what factors might explain health literacy variances in this patient population.

## 2. Method

### 2.1. Participants

The study participants (ages 18–81 years old; *M* = 43; *SD* = 17) are from a larger project that focuses on Deaf adults’ learning of health information online. Study protocols and further details are published elsewhere [27,28]. The participants included in this study’s analysis (*N* = 853) are those who completed the NVS or ASL-NVS. The Deaf ASL signers included in this analysis were those who demonstrated bilateral unaided hearing levels at severe or profound on a Shoebox audiometer [29–31], self-reported aided hearing levels remaining at severe hearing loss or worse, and preferred language of ASL (either monolingual ASL or bilingual ASL with English). The hearing English speakers included in this analysis were those who demonstrated no evidence of moderate or worse hearing loss on Shoebox. See Table 1 for the Deaf and hearing groups’ demographic information.

### 2.2. Materials

#### 2.2.1. Background interview

Questions were asked about the participant’s age, gender identity, ethnicity, race, income, education, employment, language, and Deaf community involvement.

#### 2.2.2. Hearing Levels

Participants’ unaided hearing levels were tested using a Shoebox audiometer [29–31]. The tablet-based interactive app required participants to touch an object on the screen which then either makes a sound or not. There are two bins on the screen, one for objects that make sounds and one for those that do not, and participants drag the object in their appreciated bin. Sometimes the object does not make a sound, but at other times the sound might be below the participant’s hearing threshold. The object continues to appear on the screen until multiple frequencies and intensities are evaluated providing audiograms of warble-tone thresholds in four frequencies (500, 1000, 2000 and 4000 Hz). The use of the Shoebox audiometer was used to determine eligibility determination and reducing the potential for sample bias due to misclassification. By objectively confirming severe or profound hearing loss, we minimized the inclusion of Deaf signing participants with milder hearing loss who may still use ASL, helping to ensure the groups truly reflect the characteristics under investigation. Deaf signers with milder forms of hearing loss may be able to access information by listening (e.g. radio, spoken information). Likewise, the audiometer helped to exclude any hearing participants who may have had more severe forms of hearing loss (moderate or worse) that can lower their ability to access information by listening.

#### 2.2.3. General cognitive ability

To ensure that the Deaf and hearing groups were similarly assessed for their cognitive ability, we administered the *Matrix Subtest* of the *Kaufman Brief Intelligence Test* [32], a language-free (non-verbal) visual test. This subtest measures the ability to solve new problems, perceive relationships, and complete visual analogies without testing vocabulary or language skills. Participants are shown pictures or abstract designs that follow a pattern but are missing one element and are asked to point to the multiple-choice picture that would complete the pattern. Standard scores from the test’s age matched normative sample were used in the study’s analyses [33–35]. This test was selected because it does not require the use of speech or spoken instructions, making it ideal for deaf participants, and it has been used in past studies with Deaf individuals.

#### 2.2.4. Health literacy

**Fig. 1.**
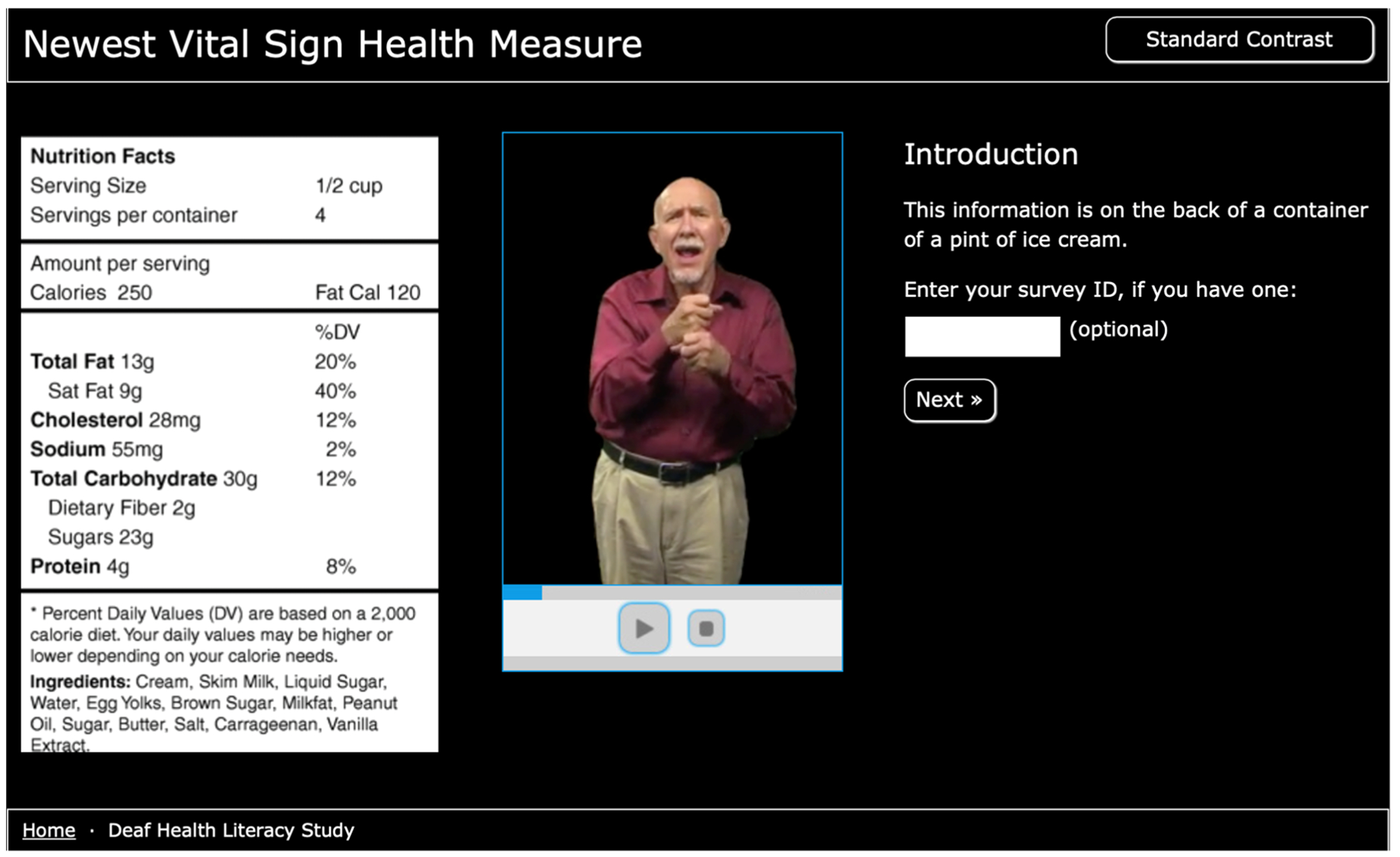
Screenshot of Newest Vital Sign in American Sign Language Version.

The study used an adapted, translated, and validated health literacy instrument for Deaf signers, the ASL-NVS [23], which assesses a person’s ability to answer six questions about a nutrition label. The ASL-NVS is the first, and currently only, health literacy instrument available for Deaf signers (see Fig. 1). The ASL-NVS and the original English version of the NVS were used to assess the health literacy score in both Deaf and Hearing participants, respectively.

#### 2.2.5. Language proficiency

ASL and English Language proficiency was measured by sentence reproduction tests as they are sensitive to developmental proficiency of first language [36,37] and second language learners [38–40]. The Deaf participants took the *American Sign Language Sentence Reproduction Test* (ASL-SRT) [41] that required them to watched videos of 20 ASL sentences and were tasked to reproduce the sentences after each one was presented. Correct reproductions are awarded 1 point, and the maximum total score is 20. The Hearing participants took the *Speaking Grammar Subtest of the Test of Adolescent and Adult Language, Third Edition* (TOAL3) [42], a parallel test that the ASL-SRT was modeled after, that assesses spoken English proficiency. There are 30 English sentences, and correct reproductions are also awarded 1 point, and the maximum total score is 30.

#### 2.2.6. English reading grade equivalent

The *Test of Silent Contextual Reading Fluency-2* (TOSCRF-2) [43] was selected as the measure of English reading skills because, unlike many reading tests, it does not require hearing or speaking abilities. Participants are given a word chaining test that measures word identification, word comprehension, and reading speed. The test consists of 220 un-related words printed in rows with no spaces between them, beginning with pre-primer level words and increasing in difficulty to adult-level words. Participants are asked to draw lines to separate as many recognizable words as possible within three minutes (e.g., lightwhatpinkplant results in a divided chain of light|what|pink|plant). Grade equivalent scores derived from the test’s adult normative sample were used in analyses.

### 2.3. Procedures

Participants were recruited by flyers, social media, announcements at social clubs, and booths at community events at three different metropolitan areas: Chicago, Illinois (143 Deaf, 147 Hearing), Flint, Michigan (139 Deaf, 148 Hearing), and Rochester, New York (126 Deaf, 150 Hearing). Participants were tested (2017–2020) individually by staff research assistants trained in the study protocol who were fluent in the language (English or ASL) used by the participant. Participation in the larger project took up to 2 h, and participants were compensated $40. All data was stored in REDCap. The procedures were approved by the University of Michigan’s and Rochester Institute of Technology’s Institutional Review Boards for human subject protection.

### 2.4. Analytic approach

Participants’ demographic variables and performance on study measures that had means were analyzed using *t*-tests with 95 % confidence intervals (CI) lower and upper limits, and those that had percentages were analyzed using Chi-square tests to determine if there were any ASL and hearing group (independent variables) differences based on background characteristics. NVS scores were dichotomized and coded as *Adequate Health Literacy* (Raw Score of 4–6) and *Inadequate Health Literacy* (Raw Score of 0–3), which was the main dependent variable of this study. Odds ratios were computed with Group (Deaf and Hearing) and NVS Category (Adequate and Inadequate). Stratified health literacy comparisons were computed between Deaf and Hearing patients using Chi-square for Hispanic and Race groups. Due to predicted health literacy adequacy differences among Deaf signers compared to their hearing counterparts across racial and ethnic groups, race and ethnicity were not put into the model since it would be interpreted as a confounder. To determine the amount of variance in health literacy accounted for by language proficiency as measured by the ASL-SRT (Deaf group) and TOAL3 (Hearing group) and reading ability grade equivalent (TOSCRF-2), binary logistic regressions were performed for the Deaf and Hearing groups separately because the two groups had different language proficiency measures. Language proficiency and reading ability were inserted and considered as predictor variables in the model. Nagelkerke *R^2^*values—sometimes referred to as pseudo *R^2^* values when used with binary data—and category predictions were reported along with odds ratios for the variables in the equations. IBM SPSS Statistics version 28 was used for all analyses.

## 3. Results

The Deaf participants, compared to the Hearing participants, were older and proportionally more Hispanic and White (Table 1). There were no Deaf (*M* = 95.6; *SD* = 18.7) and Hearing (*M* = 96.7; *SD* = 18.1) group differences based on general cognitive ability (Mean difference = 1.1 95 % CI: −1.10, 1.28). Deaf participants had significantly lower English reading grade level (*M* = 7.7; *SD* = 3.5) than Hearing participants (*M* = 9.4; *SD* = 3.6). The odds of having inadequate health literacy among Deaf patients was 3.7 times higher (95 % CI: 2.76, 4.90) than that of Hearing patients. Inadequate health literacy was more likely among Deaf participants who identified as Non-Hispanic White (*p* = <.001), Non-Hispanic Patients of Color (*p* = .022), and Hispanic Patients of Color (*p* = <.001) (Table 2) compared to their Hearing counterparts with similar racial and ethnic identities.

A logistic regression model among Deaf signers, including ASL proficiency and English reading grade level, on the likelihood of having adequate health literacy, was statistically significant, χ^2^(2) = 101.52, *p* < .001 and explained 34.2 % (Nagelkerke *R^2^*) of the variance in health literacy. Deaf signers who were more fluent in ASL (ASL-SRT *M* = 6.07; *SD* = 4.07) had a higher likelihood of having adequate health literacy (Odds Ratio = 1.10, 95% CI = 1.02, 1.17; *p* = .009). Similarly, Deaf signers who had a higher English reading grade level (TOSCRF-2 *M* = 7.76; *SD* = 3.56) also had a higher likelihood of having adequate health literacy (Odds Ratio = 1.40, 95 % CI = 1.28, 1.53; *p* = <.001). Among Hearing participants, using the TOAL3 instead of ASL-SRT, the model was statistically significant, χ^2^(2) = 171.07, p < .001. The model explains 47.8 % (Nagelkerke *R^2^*) of the variance in health literacy. Hearing adults who were more fluent in English (TOAL3 *M* = 18.84; *SD* = 2.78) also had a higher likelihood of having adequate health literacy (Odds Ratio = 1.39, 95% CI = 1.24, 1.57; *p* = <.001), and those who had a higher English reading grade level (TOSCRF-2 *M* = 9.41; *SD* = 3.63) also had a higher likelihood of having adequate health literacy (1.40; 95 % CI = 1.29, 1.51, *p* = <.001).

## 4. Discussion

Deaf patients had a 3.7 times higher risk, compared to Hearing patients, for inadequate health literacy. This study is the first to demonstrate that Deaf patients with greater ASL proficiency demonstrated higher health literacy. The instruction of ASL is often not encouraged by hearing health care providers who work with parents of Deaf children because of the long-standing myth that it might harm their ability to learn spoken and written English [44]. Most Deaf infants and toddlers in the USA are language deprived, at least during the first couple of years of their lives where they are not taught or exposed to ASL [45]. The development of ASL proficiency among those with delayed language exposure is often limited in comparison with ASL signers who learned from Deaf parents from the earliest stages of language development [41, 46]. Most Deaf children in primary and secondary classrooms who have a teacher who signs or a sign language interpreter, are often working with professionals who learned the language as a second language during college. These teachers and sign language interpreters are often taught White-based ASL that does not reflect linguistic variations used by the diverse ASL community [47–49]. An additional challenge for Deaf signers is that they typically need a second language, English, to navigate life in the United States. Their access to their second language without difficulty (assuming no vision challenges) is often in the written format only. It was not surprising to find that English reading skills explained 8 % of health literacy variance–similar to the McKee et al. (2015) NVS [23] results with Deaf adults but confirmed here with a different reading assessment.

Hearing patients’ proficiency in spoken English better predicted their health literacy than what was seen with ASL for Deaf patients’ health literacy. This is likely explained by the scarcity of health-related information in ASL [23,27,45]. Deaf patients who use ASL to communicate often experience reduced opportunities for incidental learning in a variety of settings (e.g., schools, health care, and work) [23]. A phenomenon called “dinner table syndrome” encapsulates the frequently missed learning opportunities among many Deaf individuals [13]. Deaf individuals watch their family members and friends converse with each other but may be unable to decipher what is being said. These informal discussions yield rich and helpful health information (e.g., family history) for hearing individuals but remain unavailable for Deaf signers. This is further exacerbated by communication breakdowns between clinicians and Deaf signers. With sub-optimal access services or lack of accommodations, the effect on Deaf signers’ health might be worsened. Although the use of interpreters [16,22] is the standard of care for Deaf signers, they are infrequently provided [16,17,50]. A significant number of ADA related lawsuits stem from failures to ensure effective communication in healthcare. In a study led by Iezzoni, over a third of clinicians reported not knowing their legal responsibilities under the ADA [51]. Providing clinicians with better training and involving patients in choosing the appropriate accommodation can improve accessibility and reduce legal risks especially with their Deaf signing patients [16,22,52]

To overcome scarce health information in ASL, many Deaf signers learn language, health information, and even culture via peers rather than family, and struggle to identify and correct misinformation [53]. The accumulation of inaccessible health information, clinician-patient communication breakdowns, loss of incidental and formal learning opportunities and inadequate health literacy may be an important cause of the lower level of health-related knowledge and worse health outcomes that have been observed among Deaf individuals [22,50,54–58]. Prior health information sources in ASL have not been sustained due to limited funding. This included the loss of sites such as DeafMD.org and the ASL video library previously available on Medline Plus [59]. Future efforts and funds are much needed to provide Deaf signers with a centralized health library in ASL.

Inadequate health literacy among Deaf signers is a public health concern, indicating that Deaf patients may need additional time for education and counseling during clinical visits. Previous efforts to integrate educational programs into clinical settings did not reveal specific barriers for individuals with limited health literacy [60]. However, for Deaf signers, ensuring accessible language and communication is essential before implementing any health literacy interventions [22,61]. When communication became accessible, either through an interpreter or an ASL fluent clinician, Deaf signers identified clinicians as a major source of trustworthy health information [27,53, 62].

Clinicians and healthcare systems should routinely assess all patients’ language and communication preferences to avoid assumptions and ensure that necessary accommodations, such as interpreters for Deaf patients, are provided proactively [16,22]. Existing disability screeners offer opportunities to standardize this approach and help make health care systems more proactive in meeting the needs of Deaf signers [63, 64]. Additionally, health literacy programs should expand to include diverse languages and communication modalities, offering better support to Deaf communities [65]. Finally, clinicians can enhance patient understanding by using strategies like the teach-back method to confirm comprehension [11].

The results of this study support the findings of other studies demonstrating disproportionally more People of Color with inadequate health literacy [3,25,26], regardless if they are Deaf or Hearing. There are compounded risks of inadequate health literacy for people who are both Deaf signers and People of Color. This suggests overlapping of social identities that interact and cumulatively magnify experiences of marginalization, discrimination, or privilege—adversely affecting health literacy among Deaf signers with Black and other intersectional identities. There is scarce research on effective strategies for addressing intersectional factors among Deaf People of Color. Potential targeted approaches include increasing access to sign language interpreters and clinicians who are both sign language fluent and identify as People of Color, as well as developing more patient education materials and videos specifically tailored for this population [66,67].

### 4.1. Limitations

The Deaf group was not as racially diverse as the hearing group, although the team attempted to recruit as many People of Color as possible. Overall, the racial diversity among Deaf participants in this study was relatively similar to the demographics of other Deaf samples in prior published studies and nationally representative datasets [68–72]. The health literacy of Deaf signers who were found to have moderate or better hearing levels by audiometry and people who report to be hard-of-hearing were not the objective of this study and not included. The characteristics and predictors of their health literacy may be different from the Deaf signers in this study and can be reviewed elsewhere [73]. Despite these limitations, this is the first known study that assessed how language proficiency and reading ability influence health literacy adequacy among Deaf signers and how these influences may differ from hearing English speaking participants.

### 4.2. Conclusion

Deaf signers had a markedly higher risk for inadequate health literacy in comparison to hearing participants. This risk was compounded among Deaf signers from racial and ethnic minority groups. Further, ASL proficiency and English reading skills positively predicts health literacy among Deaf signers. Health education needs to be accessible to Deaf signers, starting when they are in school. The use of sign language concordant clinicians and/or interpreters is also critically important to address the risk of inadequate health literacy on the health of Deaf signers.

## Figures and Tables

**Table 1:** Descriptive statistics for demographic variables by group.

*n*	Deaf 408	Hearing 445	Group Difference
Age	*M* = 46.9	*M* = 41.0	*t*(849) = 5.948, *p* = <.001
	*SD* = 16.7	*SD* = 16.8	Cohen’s d = .355 95 % CI.219,.490
Gender Identity			χ^2^(2, 853) = 2.299, *p* = .317
Male	43.3 %	39.7 %	
Female	56.3 %	60.2 %	
Other	0.2 %	0.0 %	
Ethnicity			χ^2^(1, 850) = 8.189, *p* = .004
Non-Hispanic	85.7 %	91.6 %	
Hispanic	13.9 %	7.8 %	
Race			χ^2^(1, 856) = 67.862, *p* = <.001
White	71.2 %	43.3 %	
Patient of Color	28.7 %	56.6 %	
Black	12.4 %	46.4 %	
Asian	4.0 %	2.9 %	
Pacific Islander	0 %	0.2 %	
American Indian	4.0 %	1.1 %	
Other	7.7 %	5.9 %	
Income			χ^2^(3, 840) = .989, *p* = .804
> $25,000	42.6 %	42.4 %	
$25,000 - $50,000	10.1 %	6.5 %	
> $50,000	35.2 %	39.3 %	
Education			χ^2^(3, 887) = 1.333, *p* = .721
<High school	7.3 %	6.7 %	
High school or GED	24.2 %	23.3 %	
1–3 Years of college	32.5 %	36.4 %	
4 or more years of college	35.2 %	33.2 %	
Employment			χ^2^(1, 851) = .241, *p* = .241
Employed	47.0 %	45.3 %	
Unemployed^1^	52.6 %	54.3 %	
Age Deaf			
Born Deaf	60.0 %		
Deaf before age 1 year	8.8 %		
Deaf after age 1 year	29.0 %		
Unknown	2.2 %		
Deaf Caregivers/Parents			
1 + Deaf caregiver	18.5 %		
No Deaf caregivers	81.5 %		
Deaf Education			
Deaf classroom only	24.4 %		
Deaf and hearing classrooms	41.2 %		
Hearing classrooms only	34.4 %		
Social Groups			
Deaf friends only	45.5 %		
Deaf and hearing friends	52.8 %		
Hearing friends only	2.2 %		

Note:

1 Unemployed group was made up of those who reported being out of work, homemaker, student, retired, or unable to work.

**Table 2:** Adults with adequate health literacy by group, ethnicity, and race.

	Deaf	Hearing	Group Difference
Non-Hispanic White	37.2 %	91.9 %	χ^2^(1, 430) = 144.126, *p* = <.001
Hispanic White	36.8 %	66.6 %	χ^2^(1, 40) = 3.558, *p* = .059
Non-Hispanic Patients of Color	22.8 %	36.9 %	χ^2^(1, 315) = 5.280, *p* = .022
Hispanic Patients of Color	10.5 %	61.5 %	χ^2^(1, 51) = 14.009, *p* = <.001
